# Using Microscale Thermophoresis to Characterize Hits from High-Throughput Screening: A European Lead Factory Perspective

**DOI:** 10.1177/2472555217744728

**Published:** 2018-02-20

**Authors:** Julie M. Rainard, George C. Pandarakalam, Stuart P. McElroy

**Affiliations:** 1 European Screening Centre Newhouse, Biocity Scotland, University of Dundee, Newhouse, UK

**Keywords:** drug discovery, high-throughput screening, biophysical assays, microscale thermophoresis, assay development

## Abstract

High-throughput screening (HTS) is a proven method for discovering new lead matter for drug discovery and chemical biology. To maximize the likelihood of identifying genuine binders to a molecular target, and avoid wasting resources following up compounds with unproductive/nonspecific mechanisms of action, it is important to employ a range of assays during an HTS campaign that build confidence of target engagement for hit compounds. Biophysical methods that measure direct target/compound engagement have established themselves as key techniques in generating this confidence, and they are now integral to the latter stages of HTS triage at the European Lead Factory (ELF). One relatively new technique that the ELF is using is microscale thermophoresis (MST), which measures the differences in rate of movement through a temperature gradient that are caused when single molecular species form complexes. Here we provide an overview of the MST assay development workflow that the ELF employs and a perspective of our experience to date of using MST to triage the output of HTS campaigns and how it compares and contrasts with the use of other biophysical techniques.

## ハイスループットスクリーニングにおいて、ヒットの特性を把握するためのマイクロスケール熱泳動の使用：欧州リードファクトリーの見解

ハイスループットスクリーニング（High-Throughput Screening：HTS）は創薬及びケミカルバイオロジーにおいて新たなリード物質を発見する為の確立された方法である。分子標的への純粋なバインダーを同定する確率を最大限にし、非生産的な、あるいは非特異的な作用機序をもつ化合物の追求により資源の浪費を避けるためにも、HTSキャンペーン中に広範囲のアッセイを使用することで、ヒット化合物の標的関与の信頼性をより確固としたものとすることが重要である。標的と化合物の関与を直接測定する生物物理学的手法はその信頼性を高めるための主要な技術として確立されており、現在は欧州リードファクトリー（European Lead Factory：ELF）においてHTSトリアージの後期段階には不可欠な技術となっている。ELFが使用している比較的新しいテクニックの一つに、マイクロスケール熱泳動（Microscale Thermophoresis：MST）があるが、これは単一分子種が複合体を形成する際に生じる温度勾配による移動速度の差異を測定するものである。ここでは、ELFが採用しているMSTアッセイ開発のワークフローの概要、及び、これまでにHTSキャンペーンにおいてのアウトプットをトリアージするためにMSTを使用した我々の経験についての見解と他の生物物理学的手法の使用と比較・対比して如何なる違いがあるのかを論じる。

## 마이크로 단위 열 영동을 사용하여 고속대량스크리닝에서 유효화합물 특징화하기: 유럽 리드 팩토리 전망

고속대량스크리닝(HTS)은 신약 개발 및 화학생물학에서 새로운 선도 물질을 개발할 때 사용하는 증명된 방법입니다. 분자 표적에 대한 진성 결합 물질의 식별 가능성을 최대화하고, 비생산적이거나 비특이성 메커니즘이 작용하는 화합물의 자원 낭비를 줄이기 위해서라도 유효화합물에 대한 표적 집중의 신뢰도를 높이는 HTS 캠페인을 시행하는 동안에는 분석 범위를 사용하는 것이 중요합니다. 직접 표적 및 화합물 집중을 측정하는 생물물리학 방법은 이 신뢰도를 만들어내는 주요 기술로 자리매김하였으며 유럽 리드 팩토리(ELF)의 HTS 선별 최후 단계에서 필수 요소가 되었습니다. ELF에서 사용하는 비슷한 새로운 기술로는 마이크로 단위 열 영동(MST)가 있습니다. 단일분자종이 복합체를 형성할 때 발생하는 온도 기울기의 이동속도 차이를 측정합니다. ELF가 사용하는 MST 분석 개발 흐름의 개요와 HTS 캠페인 결과물 선별에 대한 최신 MST 사용 경험에 따른 전망 그리고 기타 생물물리학 기술 사용과의 비교 및 대조를 해봅니다.

## 使用微尺度热泳来描述高通量筛选命中情况的特征：“欧洲先导化合物工厂”观点

在药物发现和化学生物中，高通量筛选(HTS)是一种成熟的方法，用来发现新的先导物质。为了尽可能识别分子靶标真正的粘结剂，避免因化合物与无效/非特异性作用机制的结合而浪费资源，在HTS活动中，务必要利用一系列的试验来建立命中化合物的靶标衔接的信心。在培养这种信心的过程中，测量直接目标/复合衔接的生物物理学方法已经确立了其关键技术的地位， 目前，在“欧洲先导化合物工厂”(ELF) HTS分类的后期阶段，它们是不可或缺的一部分。ELF正在使用一种相对较新的技术，即微尺度热泳(MST)，它通过一个温度梯度来测量移动速率中的差异，当单分子物种形成复合物时，会形成这种温度梯度。我们在这里概述了ELF采用的MST试验开发工作流，以及一个观点：我们迄今为止使用MST来对HTS活动的产物进行分类的经验，以及它与使用其他生物物理学技术的比较和对比情况。

## Using Microscale Thermophoresis to Characterize Hits from High-Throughput Screening: A European Lead Factory Perspective

Le criblage à haut débit (HTS) est une méthode de référence pour découvrir des molécules de départ qui serviront à concevoir des médicaments ou à étudier des mécanismes biochimiques. Pour maximiser la probabilité d’identifier de véritables ligands pour une cible moléculaire, et faire l’économie d’une optimisation coûteuse de composés dont les mécanismes d’action sont non spécifiques, il est important d’utiliser une gamme de tests au cours d’une campagne HTS. Les méthodes biophysiques qui mesurent l’engagement direct entre cibles et composés sont reconnues comme des techniques clés pour renforcer ce niveau de confiance, et elles font maintenant partie intégrante des dernières étapes du triage des touches issues du HTS au sein du consortium European Lead Factory (ELF). En particulier, l’ELF utilise la technique relativement récente de thermophorèse à micro-échelle (MST) qui mesure les différences de vitesse de déplacement dans un gradient de température entre les espèces moléculaires individuelles et les complexes qu’elles peuvent former. Nous présentons ici un aperçu du processus de développement des tests MST effectués par l’ELF et une perspective de notre expérience à ce jour de l’utilisation de la MST pour le triage des touches de criblage HTS, ainsi qu’une comparaison de cette méthode avec d’autres techniques biophysiques.

## Introduction

The European Lead Factory (ELF) is a major Innovative Medicines Initiative (IMI)–funded project aiming to discover and develop novel chemical structures for drug discovery programs in the public and private sectors.^1^ To achieve this aim, a Joint European Compound Library (JECL) has been established by combining approximately 300,000 mostly proprietary compounds from participating pharmaceutical companies, with up to 200,000 additional bespoke compounds designed and synthesized during the course of the project, based on contributed ideas from around Europe.^2^ European academics and small and medium-sized enterprises (SMEs) are eligible to submit target proposals to the ELF, and following a review process, successful programs are scheduled to be screened against the entire library within the European Screening Centre (ESC), primarily based in Scotland and the Netherlands. One of the major deliverables of the ELF is the provision of a qualified hit list (QHL) of 50 or fewer hit structures to the academic or SME submitter of the target. They then gain the rights to exploit the compounds on the QHL as chemical starting points for drug discovery programs or as tools for investigating and validating novel pharmacological targets. The nomination of the QHL represents an irreversible decision gate, at which point the legal rights and obligations are committed to eliminating the possibility of mining for alternative hit structures if later investigations invalidate the compounds as not developable.^3^ It is therefore of critical importance to use a range of tools and techniques in a high-throughput screening (HTS) workflow that minimizes the chance of this least desirable of outcomes.^4^

A well-designed screening workflow, also known as a screening cascade, should be bespoke for every target and consists of a series of assays through which compounds are tested that maximizes the likelihood of identifying genuine binders of the target while minimizing the risk of selecting undesirable compounds. These undesirable compounds, sometimes referred to as nuisance compounds or pan-assay interference compounds (PAINS),^5^ have been reviewed extensively elsewhere,^4–8^ with different targets/assays being sensitive to different mechanisms of interference, and include fluorescent or colored compounds that disrupt the assay readout,^9,10^ redox cycling compounds,^11^ aggregators,^12^ metal chelators,^13^ DNA or RNA intercalators,^14^ and metal contaminants.^15^ The probability of misidentifying these undesirable compounds as genuine hits is reduced by introducing deselection assays into the screening cascade, which should identify compounds exhibiting the undesirable behaviors that the target/assay is sensitive to. Such compounds are *probably* not meaningfully interacting with the target and are therefore deprioritized or dismissed from further selection, but that does not eliminate the possibility that they *may* still be meaningfully binding to the target. A more satisfactory approach is, where possible, to adopt a positive selection strategy in the form of one or more orthogonal assays that aim to confirm on-target activity, yet are different enough from the primary assay that they are likely to have a different profile of sensitivity to nuisance compounds. Most compound screens against isolated protein targets involve the use of a microplate-based biochemical assay, which is relatively cheap and reliable and can be performed on a scale required for HTS. Typically, these involve a probe acting as a surrogate of the physiological function of the target and are almost always light based, being monitored with a multilabel plate reader via some form of fluorescence, absorbance, or luminescence. Using an orthogonal biochemical assay with a probe complementary to that used in the primary assay (i.e., fluorescence rather than absorbance, ideally at differing wavelengths) is a good way of rapidly following up on large numbers of hits to eliminate those that interfere with the primary assay technology. However, many nuisance compounds disrupt the protein function or structure rather than simply interfere with the signal, and so tend to be active in both assays. An alternative, complementary, and increasingly adopted approach in the latter stages of HTS is the use of biophysical assays.^16,17^ These assays probe the nature of the direct interactions between ligands and proteins rather than relying on the functional activity of the protein. Most biophysical techniques are still relatively low throughput, and therefore not amenable to primary screening, but can play a very important role in HTS triaging to confirm target engagement of hits, validate their selection for further optimization, and inform structure–activity relationships (SARs).^18–20^

## Use of Biophysics in the European Lead Factory

A wide range of biophysical methods are available to measure the affinity of ligand–protein interactions. Each technique provides a range of different information on the binding specificity, stoichiometry, kinetics, and/or thermodynamics of binding interactions, with their own advantages and disadvantages, which have been reviewed extensively elsewhere.^17–19,21^ Unlike label-based biochemical assays, most biophysical assays tend to involve fewer assay components, making them less prone to compound interference or aggregation.^19^ Many are also applicable to a wide range of target classes and are not reliant on monitoring target function, so the assessment of binding interactions does not require an enzyme to be active, or even for the function of the target to be known, and compound binding can be measured regardless of their mechanism of action (i.e., agonists, antagonists, activators, or inhibitors).

No single biophysical technique appears to be more reliable than any other as a hit triaging tool, with different methods having been shown to identify different populations of hits from the same screen.^22–24^ For this reason, if possible, a number of techniques are best used in combination to provide the most comprehensive assessment of target binding for hits from an HTS campaign. The choice of technology is driven by consideration of various factors, ranging from assay sensitivity and ligand and protein material requirements to the availability of in-house equipment and expertise (see Renaud et al. for an excellent review summarizing the pros and cons of the most commonly applied technologies^17^). The ELF manages a very busy project portfolio of >80 distinct targets and operates at a throughput of 15–20 ultra-high-throughput screens per year, meaning that there is a balance to be struck with the amount of time and resources that can be invested in each project. The portfolio of targets is diverse and represents all major target classes, consisting of enzymes (58%), transporters (1%), receptors (7%), ion channels (6%), protein–protein interactions (15%), protein–DNA interactions (4%), protein–RNA interactions (2%), and a variety of other targets with a diversity of functions (8%). This includes proteins ranging from 20 kDa up to >1 MDa in size, and often the amount of target protein for individual targets is limited, in terms of both the quantity that can be supplied and the number of different constructs that are available. We also have to operate with very stringent compound usage restrictions, whereby we are limited to 5.5 µL of 10 mM DMSO stock to follow up hits in the primary, deselection, selectivity (if required), and biochemical and biophysical orthogonal assays. Despite these limitations, we still aim to employ more than one biophysical method to identify positive hits and have adopted three complementary techniques: surface plasmon resonance (SPR), microscale thermophoresis (MST) and thermal shift assay (TSA; also known as differential scanning fluorimetry [DSF]). The strict reagent restrictions make techniques such as isothermal titration calorimetry (ITC) and nuclear magnetic resonance (NMR) impractical.

## Microscale Thermophoresis

MST is emerging as a sensitive method that can be used to assess biomolecular interactions^25–29^ and has been utilized to study interactions between a variety of binding partners of various molecular sizes: protein–protein, antibody–antigen, protein–DNA, and protein–RNA interactions and the binding of ligand to ternary complexes.^26–28^ The use of MST is increasingly being reported in fragment-based drug discovery,^23,24,30–33^ including kinases such as p38α^30^ and MEK1,^31^ and the bromodomain BRD9.^32^ In both the MEK1 study by Sanofi in collaboration with NanoTemper and the BRD9 study by Boehringer Ingelheim, MST was used to confirm the binding of fragments identified by SPR and DSF, as well as to identify additional hits not detected by the other techniques. Pollack et al. compared the binding affinity of a handful of fragments to p38α measured by MST and other assays, such as SPR, mobility shift assay, and a fluorescence lifetime assay, and found good agreement across these different technologies.^30^ Unlike fluorescence polarization (FP) or reporter displacement assays, where one might fail to identify binding to allosteric sites, as the test compounds need to compete for binding with a fluorescent tracer, MST allows the quantitative measurement of the direct binding affinity of a compound to the target, enabling the detection of binding to orthosteric and allosteric/cryptic sites.^34–36^ This may explain the broad applicability of the technique and how additional hits are identified using direct binding methods.^24^ However, if required, MST assays can be designed to perform competition experiments with a reference ligand.^26^

MST measures the interaction between biomolecules in solution. Thermophoresis, also known as the Ludwig–Soret effect, is defined as the direct movement of molecules through a temperature gradient.^37,38^ A molecule’s thermophoretic properties are determined by charge, size, and hydration shell.^27^ Upon binding to some interacting partner, one or more of these three parameters may be altered, resulting in a change in its thermophoretic movement.^26^ Measuring changes in thermophoresis can then allow for the quantification of the affinity of the interaction between the binding partners. There is the potential for complementary, conflicting, or confounding influences on the three key parameters, which makes it difficult to predict the magnitude of effect, if any, that the formation of a given complex will have on thermophoretic movement. The suitability of MST as a technique for assessing target–ligand interactions must therefore be assessed on a case-by-case basis with careful assay development. To monitor the thermophoresis of biomolecules, the Monolith instruments commercialized by NanoTemper use an infrared (IR) laser, which heats an aqueous sample loaded in a thin glass capillary. The laser focus produces a localized microscopic temperature gradient with a heat range of 2–6 °C, and ligand–target protein complexes demonstrate a different movement velocity through the temperature gradient compared with unbound molecules. This movement through the laser-heated spot is monitored via fluorescence of the target protein, either from the intrinsic UV fluorescence of tryptophan, tyrosine, and phenylalanine residues (tryptophan exhibiting the strongest fluorescence) or from labeling the protein with a fluorescent dye through covalent attachment to lysine or cysteine residues,^27^ or noncovalent attachment to a polyhistidine tag.^39^ Rapid scanning of a series of capillaries containing a constant concentration of fluorescent target molecule and increasing concentrations of ligand enables the determination of equilibrium binding constants. Alternatively, the ligand can be fluorescently labeled and the unlabeled protein titrated.

Unlike technologies such as SPR, which rely on detectable changes in size or mass triggered by a binding event, variations in the thermophoretic profile can be caused by minute changes in the target molecule’s solvation entropy, even in the absence of changes to the protein’s size or charge.^26^ Binding interactions between very small ligands and large proteins are therefore detectable, as shown by Wienken et al. with the binding of calcium ions with a molecular weight of 40 to the 16.7 kDa calmodulin protein, corresponding to a molecular ratio of 417.5.^25^ This explains why MST is gaining popularity as a technique for fragment screening, because it allows the study of weak-affinity fragments titrated up to 10 mM.^31,32^ In addition, MST is described as offering a wide detection range, from low-millimolar to picomolar affinities, when using NanoTemper’s dedicated Monolith NT.115^Pico^ instrument.^28^ In general, dissociation constants (K_d_) within twofold of the protein concentration can be accurately quantified.^27^ One of the greatest advantages of MST in an ELF context is that it works with very low amounts of ligand and dye-labeled target.^26,27^ Although the Monolith NT.Automated instrument can only accommodate 96 capillaries per run, in our experience the short measurement times of 15–25 s per capillary enable rapid screening of at least 800 samples per day. MST allows the measurement of molecular interactions in solution, avoiding the need to immobilize proteins to surfaces and reducing the observation of nonspecific compound binding to coupling chemistries, which can sometimes complicate the analysis when using techniques such as SPR.^40^ Furthermore, MST is reported to work in both simple, common buffers and more complex biological systems, such as cell lysates and serum.^27,28,41^ It can also be used with solubilized proteins and proteins in liposomes, as shown in a study on SNARE proteins.^26^

## MST as a Tool in the European Lead Factory

**Figure 1.**
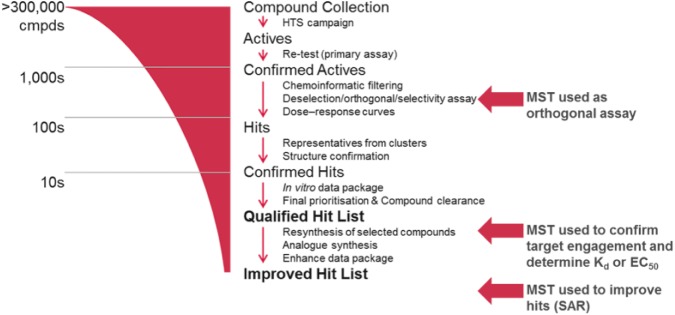
Typical screening cascade for an ELF program. Targets are screened at a single concentration of 10 µM, followed by confirmation of activity again at 10 µM. Deselection, orthogonal, or selectivity assays are used to prioritize promising hits before confirming their potency in the primary assay. A maximum of 100 compounds are selected for liquid chromatography–mass spectrometry (LC-MS) analytical assessment, and up to 55 compounds undergo a final intellectual property clearance before selection of the QHL, comprising up to 50 compounds. For promising programs, hit validation and optimization can lead to an improved hit list (IHL). MST can be used at various stages of the triage process (indicated by red arrows).

**Figure 2.**
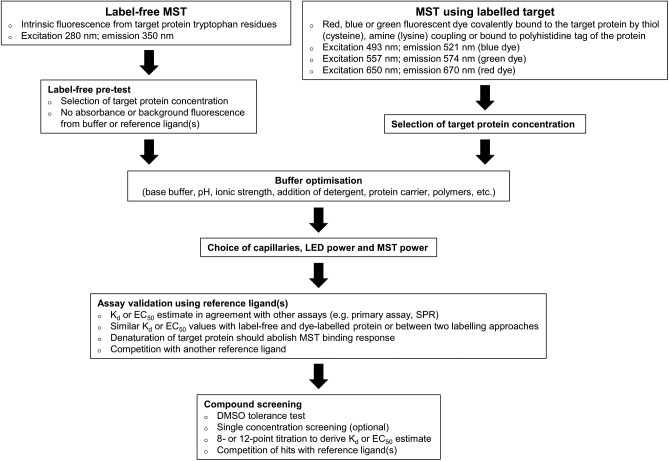
MST assay development and screening workflow.

Currently, there are no published reports on the use of MST as a triage tool for HTS, but we have found that the technology is adaptable for use at a number of different stages in the ELF screening workflow (**Fig. 1**). The low sample requirements and applicability to a wide range of proteins make MST an ideal technology when considering the diversity of the ELF portfolio and the strict sample limitations. We have used MST as an orthogonal assay to inform triage decisions and progress hits from primary screens; however, the throughput is such that we find it is not ideal for triaging many thousands of potential hits. A strategy that can be employed to increase the throughput is the testing of all compounds at a single concentration, as described for a fragment screen by Martin et al.^32^ We have investigated this approach with tens of compounds, following up the statistically significant actives as full dose–response curves, but have concerns about the high false-positive and -negative rates we observed, as will be discussed later. More typically, we use computational selection of representative hits from structural clusters, together with interesting singletons, to reduce the number of hits from a screen to the low hundreds, allowing us to test all compounds at multiple concentrations. We also use MST to provide detailed characterization of hit molecules following release of the QHL, and for the testing of new analogs synthesized by the ESC Medicinal Chemistry group for affinity ranking and providing SAR. Usefully, MST can often also identify false positives and the mechanism by which they interfere, such as photobleaching, photoenhancement, and autofluorescence (apparent in the magnitude and shape of the MST trace), and aggregation (apparent as irregular bumpy traces).^31,42^ The MST assay optimization procedure is usually rapid and the analysis of binding interactions and determination of binding affinities (K_d_ or EC_50_) simple. We have established a stepwise workflow to developing MST assays and screening hit compounds (**Fig. 2**), which involves the optimization of several key parameters. The following is a description of the workflow and how the data are interpreted and presented to the project teams.

## Choice of MST Labeling Approach and Coupling Chemistry

The first assay development step is to choose the detection method. The two main MST approaches are label-free MST, measuring intrinsic UV protein fluorescence, and labeled MST, where the protein is coupled to a longer-wavelength fluorescent dye. The Monolith NT.Automated instrument can be fitted with up to four channels to detect UV, blue, green, and red fluorescence of proteins at nanomolar concentrations or the fluorescence of red dye–labeled proteins at picomolar concentrations (if the picodetector is installed). This diversity of detectors allows for the measuring of commonly used dyes and fluorescent proteins, such as GFP, TAMRA, Cy5, and fluorescein. Our instrument is fitted with the UV detector and the nanodetector for blue and red fluorescence, which provided us with the widest range of available detection options. Any commercially sourced dye covering the requisite fluorescence wavelengths is suitable for labeling proteins. Common coupling chemistries include thiol-reactive maleimide-linked dyes for the labeling of cysteine residues and amine-reactive *N*-hydroxysuccinimide (NHS)-linked dyes for lysine labeling.^27^ Recently, a red fluorescent dye was released by NanoTemper containing a tris-nitrilotriacetic acid (NTA) moiety for noncovalent coupling to polyhistidine tags.^39^ These dyes often come as kits, including gravity-flow size-exclusion desalting columns, to buffer exchange the target protein if its storage buffer comprises components incompatible with the chosen coupling chemistry. For instance, free amine containing buffers such as Tris or glycine will compete for coupling to the NHS dye, or buffers containing high concentrations of imidazole will disrupt the binding between the nickel(II) ions of the tris-NTA dye and the protein polyhistidine tag.^43,44^

The choice between label-free (280 nm excitation, 350 nm emission) or labeled (blue: 493 nm excitation, 521 nm emission; green: 557 nm excitation, 574 nm emission; red: 650 nm excitation, 670 nm emission) MST is influenced by the project requirements. We prefer to use the red dyes, as many screening compounds and reference molecules interfere at UV wavelengths;^9^ however, our experience is that dye modification of proteins can disrupt their structure and function, sometimes limiting this option. Structural knowledge of the target protein therefore provides very useful insight. For example, cysteine labeling should be avoided for proteins with known disulfide bridges critical to their structural integrity. Similarly, reactive dyes should be used with caution for proteins containing active-site cysteines or lysines, as this may result in steric hindrance, affecting the binding of orthosteric ligands and yielding an inaccurate K_d_ value, or completely ablating binding. Some effort can be taken to vary the dye–protein stoichiometry and/or labeling time to reduce the population of active-site blocked protein molecules or try to mask active-site residues by including reference ligands during the coupling process, but our success with this technique has so far been limited.

The approach of noncovalent His tag labeling offers an attractive alternative when the ligand binding site on the protein is known to contain critical cysteine or lysine residues or has not yet been characterized. It is also a very convenient approach within the ELF because most proteins are supplied by the submitter of the target, and in the majority of cases, these proteins contain a 6xHis tag. One issue to be aware of, however, is the potential for hit compounds to appear as false positives through an interaction with the label/His tag and not a binding site on the protein. To control for this, the NanoTemper labeling kit includes a peptide with a 6xHis tag that can be labeled to assess the interaction of the ligand with the tag or tris-NTA dye. When possible, it is recommended to compare the affinity estimates of a reference ligand measured using both label-free and labeled MST approaches. When this is not an option, for example, because the label-free MST assay is too noisy or the reference compound exhibits UV fluorescence, K_d_ or EC_50_ values measured using two different labeling approaches can be compared instead. An example of this comes from a protein–protein interaction project in the ELF. The target protein was either covalently labeled to lysine residues via amine coupling or noncovalently labeled via its polyhistidine tag using the tris-NTA dye. Titration of the unlabeled binding partner yielded similar EC_50_ estimates when using both labeling techniques, which were also in agreement with the AlphaScreen primary assay.

**Figure 3.**
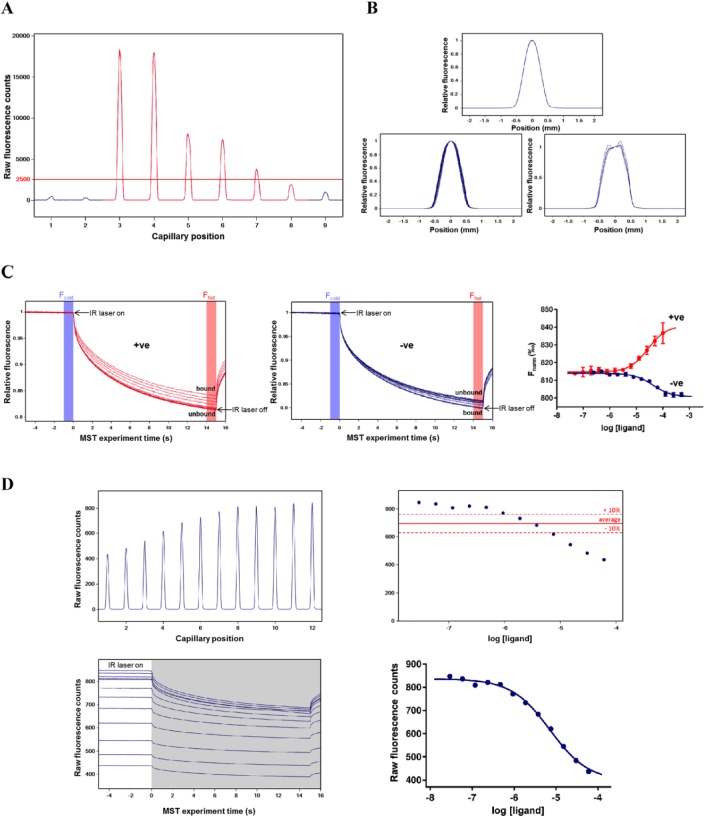
Representative data collected during MST assay development. (**A**) UV fluorescence capillary scan for label-free pretest with 500 nM (capillaries 3 and 4), 250 nM (capillaries 5 and 6), and 100 nM (capillaries 7 and 8) target protein. Protein storage buffer (capillary 2) and a high concentration of reference ligand (capillary 9) are also tested for fluorescence and compared with distilled water (capillary 1). (**B**) With no protein adsorption onto the capillaries, all capillary shape graphs overlay perfectly (top panel). Wide peaks and a lack of superimposition of the capillary shape graphs indicate unspecific protein adsorption to capillary walls (bottom left panel). Very strong adsorption of samples to the capillaries is characterized by a wide double peak (bottom right panel). (**C**) Typical MST traces for a compound showing positive thermophoresis (left panel) and a compound showing negative thermophoresis (middle panel). Compounds were titrated using a twofold dilution series starting at 500 µM (compound in blue) and 60 µM (compound in red), with 15 nM lysine-labeled target protein in buffer as recommended by NanoTemper (50 mM Tris-HCl [pH 7.4], 150 mM NaCl, and 10 mM MgCl_2_ + 0.05% Tween 20) using standard capillaries. Measurements were performed using the nanodetector with 40% LED excitation power, 40% MST power, and 5 s MST on-time. Normalized binding response (F_norm_) of compounds showing positive (red) and negative (blue) amplitudes (right panel). (**D**) Capillary scan showing compound-induced changes in protein fluorescence (top left panel). Example of a compound causing a concentration-dependent variation in fluorescence greater than 10% across a series of capillaries (top right panel). MST traces showing a compound causing a decrease in baseline fluorescence (bottom left panel in white). In such an occurrence, the baseline data should be used rather than the T jump and/or thermophoresis data (shown in grey) to assess compound binding (bottom right panel).

A key criterion to consider when deciding between labeled and label-free MST is the range of affinities that one aims to detect. When screening fragments or HTS hits with affinities in the micromolar or millimolar range, both approaches are generally applicable. However, during a hit optimization program, where compounds can demonstrate low nanomolar affinities, label-free MST is unlikely to be suitable due to the relatively high protein concentrations needed for this approach. To select a suitable concentration of target protein, fluorescence is measured for a series of concentrations (usually three are sufficient). A concentration-dependent fluorescence signal is expected, and the lowest protein concentration yielding >200 counts using the nanodetector or >2500 counts using the UV detector is required for continued assay development (**Fig. 3A**). The minimum protein concentration suitable for label-free MST depends predominantly on the number of tryptophan residues. Seidel et al. reported that a protein with an average of two or more tryptophans could be used in concentrations down to 100 nM, enabling the accurate quantification of K_d_ values equal to or greater than 50 nM.^45^ However, the location of the tryptophan residues within the protein and their local environment dictates the fluorescence efficiency, meaning that some proteins require much more than two tryptophan residues to yield a suitable fluorescence signal. In our experience, 150 nM of a protein containing seven tryptophans can provide a satisfactory signal-to-noise ratio. In contrast, we find that a concentration of dye-labeled protein of 15–50 nM is generally sufficient to provide a suitable signal, and have been able to use as low as 5 nM for some targets. It should be noted that the protein concentration optimization step is generally not needed when using the tris-NTA labeling kit, as the commonly used 6xHis tag binds one dye molecule and a 50 nM final concentration of protein yields a suitable fluorescent signal.^39,44^ This 1:1 stoichiometry and the reversible equilibrium binding nature of the dye–His tag interaction also eliminate the free-dye/purification step that is necessary for lysine and cysteine labeling procedures. For label-free MST, the pretest is carried out to select the optimal protein concentration, but the assay buffer and reference ligand should also be tested to ensure that neither absorbs nor emits fluorescence at the tryptophan wavelengths. This is because the label-free capillaries are thinner and the detector is very sensitive to interference.

## Buffer Optimization

For all assays, the buffer composition dictates the ability to detect ligand–protein interactions.^46,47^ Most common buffers are well tolerated in MST, and we ideally aim to maintain buffer consistency with the primary biochemical assay used during the HTS; however, we have observed that alterations to the buffer are sometimes required to validate our MST assays. This is because base buffer, pH, and ionic strength can all influence the thermophoretic movement of molecules and dramatically change their MST binding characteristics. Additionally, some buffer components interfere with dye fluorescence and will need to be removed or replaced. These buffer alterations must be conducted carefully and not overly compromise the known structure and function of the target, such as the requirement for magnesium or manganese ions for ligand binding to kinases or the presence of cofactors or nonprotein organic coenzymes, such as NADP^+^, NAD^+^, or coenzyme A, necessary for certain enzymes, such as oxidoreductases.^47^ Similarly, if the target protein naturally forms disulfide bonds between cysteine residues, the inclusion of reducing agents such as beta-mercaptoethanol, tris(2-carboxyethyl)phosphine (TCEP), dithiothreitol (DTT), or l-glutathione (GSH) should be avoided, whereas for cytosolic proteins the presence of reducing agents is mostly beneficial in maintaining a reducing environment to avoid the formation of nonnative disulfide bridges, which can lead to protein misfolding.^48,49^ Detergents such as Tween 20 and Pluronic F-127 have been shown to greatly increase the UV fluorescence of MST samples, not due to intrinsic fluorescence, but rather by preventing a reduction in the in-solution concentration due to adherence to the sample capillaries or the plasticware the samples come into contact with prior to capillary loading.^50^ This can be further mitigated by using microtiter plates with nonbinding surfaces and capillaries that are coated with polymers that minimize protein sticking. The use of detergents, however, should be carefully assessed during the label-free pretest, as they can introduce background UV fluorescence, which can adversely affect the assay. In our experience, Tween 20 causes a slight increase in UV background, but NP40 detergent should be avoided completely, as it saturates the UV detector. The addition of polymers such as 0.1% PEG-8000 can prevent protein adsorption and sample aggregation, and a low concentration of reducing agent can also help prevent sample aggregation. Carrier proteins like bovine serum albumin (BSA) cannot be used for label-free MST, as BSA contains tryptophan residues and greatly interferes with the signal. It can be used for labeled MST, but one needs to be cautious in assessing the potential effects of BSA on the binding response. Indeed, Scheuermann et al. observed an artifact in the presence of 0.01% BSA characterized by a reversal in the direction of the sample’s thermophoresis at micromolar concentrations of a partner protein, as well as a concentration-dependent decrease in raw fluorescence of the labeled target protein. It seems that rather than acting as a passive carrier, BSA was interacting with the titrated protein partner to form another population of binding complexes.^50^

We presently conduct a limited buffer optimization, initially comparing the buffer recommended by NanoTemper (50 mM Tris-HCl [pH 7.4], 150 mM NaCl, 10 mM MgCl_2_, and 0.05% Tween 20) with that used for the primary HTS assay. We then assess the effect of adding/removing detergents and adjusting a couple of key components, such as salt and the reducing agent concentration. If we find that an alternative buffer composition shows better MST binding responses, we test this buffer in the primary assay to check whether it has compromised the activity of the target protein. If the assay remains active, we then retest the hit compounds in the biochemical assay to check if the altered buffer has any effect on their activity before testing them in MST. Looking to the future, we aim to develop a more comprehensive process for identifying the optimal MST buffer for each target. This would consist of experiments comparing the effects of pH, salt concentration, detergents, reducing agents, and protein carriers, separately or in combination. The significant complexity that this multifactorial approach introduces is not best tackled in a traditional linear experimental fashion, that is, adapting one variable per experiment. Rather, a more efficient design of experiments (DoE) approach would be preferable, whereby powerful statistical software and liquid handling automation are employed to create a matrix of varying buffer conditions and the data are analyzed for a maximal signal. This then facilitates iterative design of follow-up experiments to rapidly establish the optimal conditions. For a more in-depth review of DoE, see Tye.^51^

## Choice of Capillaries, LED Power, MST Power, and Data Analysis

It is important to check that the protein and reference ligands do not adsorb to the walls of the sample capillaries, as this adversely affects the MST signal and can reduce or ablate apparent ligand binding due to loss of material. This is generally assessed for protein alone in an initial capillary shape scan (**Fig. 3B**). The capillaries are available as either standard or premium, the latter of which are coated with a proprietary polymer surface that can greatly minimize protein or ligand adsorption to the capillary walls. We typically start by assessing the standard capillaries, and if there is significant protein adsorption after attempting to optimize the buffer, then we assess the premium-coated capillaries. Care should be taken, as sample adsorption can change upon addition of ligand, especially if the ligand is a protein or peptide with the potential to adsorb onto the capillaries when titrated at high concentrations.

There are two significant variables that can be manually adjusted on the MST system, LED power and MST power. The adjustment of these two variables can have very significant impacts on the ability to observe target–ligand binding and the data quality, so they must both be carefully assessed. Increasing the excitation power of the LED light source increases the sample fluorescence, which is useful in reducing the protein concentration but must be balanced against the potential for photobleaching. Increasing the MST power, corresponding to the IR laser power, increases the temperature gradient from 2 to 6 °C. At higher MST power, the fluorescence decrease and thermophoretic movement are usually greater, and this greater signal change can aid in the resolution of binding events. Increasing the MST power must be considered carefully, as excessive exposure of the biomolecules to focused heat can lead to differences in sample density and convective flow, characterized by aberrant MST traces with a fluorescence increase toward the end of the measurement time. To some extent, this is mitigated against by alterations in the period during which the IR laser is turned on, also called the MST on-time, which is shorter at high MST power (only 2 s vs. 5 s for medium MST power and 20 s for low MST power), but good practice is for low or medium MST power to be used where possible and high MST power only attempted if the binding response displays a very low signal-to-noise ratio. Regardless of the MST power, the MST measurement time should be set to at least 10 s to allow for the detection of aggregation.

In a typical MST measurement, a 5 s fluorescence baseline is first measured. Upon turning on the laser, IR radiation is absorbed by the water molecules, producing an immediate increase in temperature and a rapid quenching of fluorescence (T jump). This is followed by the thermophoresis phase, typically lasting 10–20 s, where the fluorescent molecules diffuse away from (positive thermophoresis) or toward (negative thermophoresis) the heat focus. The direction and rate of thermophoresis are dependent on the properties of the fluorescent molecules, meaning that the ligand–protein complex moves faster or slower through the temperature gradient than the unbound protein. Switching off the laser leads to an inverse T jump and reequilibration of the sample (**Fig. 3C**). To calculate equilibrium binding constants, the MST trace of each capillary is analyzed for the difference between the baseline fluorescence, also referred to as F_cold_, and the fluorescence level during the T jump or thermophoresis phases, known as F_hot_. The normalized fluorescence (F_norm_) is defined as F_hot_/F_cold_ and expressed as parts per thousand. F_norm_ values are plotted against the ligand concentration in a concentration–response curve to provide an estimate of affinity (**Fig. 3C**). Binding curves can also be displayed using ∆F_norm_ (baseline-corrected normalized fluorescence) or fraction bound (where all ∆F_norm_ values are divided by the curve amplitude) to account for differences in baseline fluorescence or response amplitude.^26,42^ Two models are available to fit the data in NanoTemper’s MO.Affinity Analysis software: a K_d_ model for binding interactions with a predicted 1:1 stoichiometry and a Hill model to fit multivalent interactions with higher stoichiometry or interactions with known cooperativity and a slope coefficient different from 1. This model will derive an EC_50_ estimate, rather than a K_d_. The MST analysis routine described above should only be applied to data where the ligand does not directly influence the baseline fluorescence of the protein. When the ligand causes >10% variation in protein initial fluorescence, then as long as the ligand is not itself fluorescent or acting as a quencher (i.e., the ligand is causing conformational changes in the protein that change the exposure of fluorescent residues or labels), direct analysis of the baseline fluorescence can be used to estimate affinity (**Fig. 3D**).

## Assay Validation with Reference Ligand

A critical assessment of the suitability of an MST assay to be used to follow up hits from an HTS campaign is that the affinity estimates of known reference ligands are in agreement with those obtained using other techniques, particularly the primary screening assay. Most hits from HTS exhibit affinities in the micromolar range and are typically tested at maximum concentrations of 10–30 µM. The consequence of this will be that a technique that demonstrates a reduction in sensitivity of as little as twofold will be unlikely to confirm the activity of many of the hits. The ideal situation is to have a collection of structurally diverse small-molecule reference compounds with varying affinities against the target. This would best model the situation of testing a diverse set of hits from HTS. In reality, many ELF targets do not have a selection of small-molecule references, particularly for novel targets aiming to identify tool compounds for the first time. If no compounds are available, then alternatives are typically sought to act as reference ligands, such as enzyme substrates, peptides, or nucleic acid oligomers. As mentioned, multiple reference ligands with varying affinities are preferred to ensure that the MST assay not only exhibits a sensitivity similar to that of other techniques, but also detects a similar rank order of affinity. More often than not, however, only one or two reference ligands are available. Each reference molecule is checked for quenching or autofluorescence by testing the highest concentration of compound on its own. Alternatively, absorbance and fluorescence spectral scans are performed using a monochromator on a multilabel reader, a cheaper and quicker alternative method that we use to prescreen large numbers of hit compounds. Additionally, a simple binding test is carried out on a single concentration of reference ligand in the presence and absence of protein to determine if the bound and unbound ligand complexes display an acceptable signal-to-noise ratio greater than 3. A full titration of the ligand then provides the affinity of the interaction. Importantly, the titration of the reference ligand should not lead to an increase in sample adsorption to the capillaries, since this can be mistaken for a concentration-dependent increase in signal. It is therefore important to check the capillary shape overlays for signs of sample adsorption. Once the samples are loaded into the capillaries, we usually measure the MST signal at different time points, immediately after the sample is loaded and then again after 30 and 45–50 min. This is to ensure that the binding has reached equilibrium and the affinity will not be underestimated, that there is no change in capillary adsorption over time, and that the signal from the binding complex is stable and consistent across the 45–50 min period required to screen at the maximum capacity of 96 capillaries. The estimated K_d_ or EC_50_ values for the reference ligands should be in agreement with reported data and, importantly, similar to those obtained in the primary assay.

A denaturation test is often used to provide evidence that the reference ligand–target MST binding response is specific to structurally intact protein. For this, the target is denatured in a 2% sodium dodecyl sulfate (SDS) and 20 mM DTT solution and heated to 95 °C. As mentioned previously, for targets labeled with the His tag dye, a His-peptide control is used to check for interference of dye–tag binding. Another control experiment for confirming binding specificity is to titrate the ligand against a protein it should not interact with, such as a version of the target where key binding site residues have been mutated, or an unrelated protein. Unfortunately, due to the limitations in protein construct availability, we do not usually have access to a mutated version of the target protein for the ELF projects. When more than one reference molecule is available, and they are known to bind in the same region of the target, then competition experiments are a very useful tool. Alternatively, titrating the unlabeled version of the target protein to compete for reference ligand binding to the labeled protein can also be quite informative.

## Compound Screening

When working with ligands or test compounds dissolved in organic solvents, it is important to determine what effect solvent has on the assay. Our compounds are stored in 4 or 10 mM stock solution in 100% DMSO. DMSO can reduce protein stability and/or functionality, so it is important to ensure that the final DMSO concentration in test samples will not have a detrimental effect on the protein or on the quality of the data generated.^52,53^ We usually test compounds at a top concentration of 40 or 100 µM (4–10 times the primary screening concentration) and aim to keep the final DMSO concentration below 5%. A DMSO tolerance test on the labeled target protein indicates the maximum concentration suitable for testing and involves comparing the MST data for three to four capillaries with labeled protein only versus labeled protein plus varying concentrations of DMSO. All quality parameters are compared to ensure that the DMSO does not have a negative effect on the target protein fluorescence (F_norm_), quality of the MST traces, surface adsorption, photobleaching, or sample aggregation. In some cases, the presence of 1% or 2.5% DMSO actually leads to an increase in thermophoretic movement.

Depending on the number of compounds being tested, the amount of available reagents, and the stage in the triage process, compounds are tested in MST as either a single concentration (usually in triplicate capillaries to provide three technical replicates) or as a full titration comprising either 8 or 12 concentrations, which allows for two to three compounds per capillary chip. For single-concentration testing, MST data are analyzed relative to positive (e.g., a concentration of reference ligand yielding saturation in a full binding curve) and negative (sample with DMSO only) controls. As mentioned previously, different ligand–protein complexes can produce both negative and positive thermophoresis. Because of this, we do not apply double referencing to the negative and positive controls; rather, we apply a statistical differentiator of three standard deviations on either side of the mean negative control only. The hits are then tested in a full 8- or 12-point titration. It is important to apply a consistent data analysis routine across all of the hits, for example, using the same F_cold_ and F_hot_ regions of analysis and the same curve-fitting algorithm for all MST traces. In addition to the quantitative data, we also employ a qualitative scoring system to classify the compounds screened by MST. This is useful to inform triage decisions, as an affinity estimate (K_d_ or EC_50_) cannot always be determined for weak binders, and it allows an expert user assessment of the quality of the data to be captured in our database. A similar scoring system, which ranges from 1 to 6, is used when assessing SPR and TSA data. The score is a qualitative assessment of the likelihood of binding, with compounds scoring 1 if they produce data similar to or better than the reference molecule. Compounds with scores of 2, 3, and 4 present a sliding scale whereby there is more uncertainty of the response, less potency, more noise, or reduced amplitude. Compounds scoring 5 display bumpy and noisy traces that are characteristic of aggregators, and if no indication of binding is observed, then the compound scores a 6. As our data scoring is subjective, we have introduced a system whereby at least two scientists independently score the data and then discuss their results. Due to the irreversible nature of our triage process, if there is disagreement between the two, then a compromise score is accepted or a third scientist breaks the deadlock.

## ELF Experience

**Figure 4.**
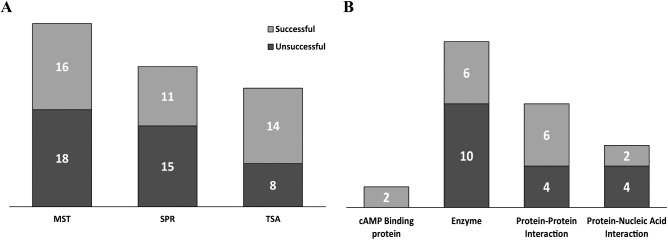
Success rates for developing biophysical assays. (**A**) Developing MST, SPR, and TSA exhibits different rates of success. (**B**) Number of successful and unsuccessful MST assays developed according to their target class.

As mentioned previously, the ELF portfolio is diverse and consists of targets from many well-known target classes, as well as from many unprecedented target classes. The targets cover many different molecular weights, structures, and functions, and where possible, we attempt to establish at least one predictive biophysical assay in the form of MST, SPR, or TSA. These three technologies have been acquired over time, so many targets have not been tested against all three, and indeed, for some targets we do not attempt one or more of the assays, as we deem them inappropriate, for example, very large molecular weight targets with SPR. That said, some interesting observations arise from analyzing the differing assay development success rates across the three technologies, success being defined as establishing a robust, reproducible assay validated with known reference ligands and capable of screening hit molecules from the HTS (**Fig. 4A**). We observe similar success rates with MST and SPR, which may seem low at 47% and 42%, respectively, but this is bearing in mind that the proteins are principally provided by the target owner as an aliquot of the screening batch, which has been produced with the biochemical assay as the main focus. These are often provided in inappropriate storage buffers, not compatible with dye labeling or immobilization procedures, and require buffer exchange with associated freeze–thawing cycles. Additionally, the reference molecules are sometimes poorly validated in the literature or the target owner’s hands or appear to be nonspecific binders, such as aggregators or denaturants, when we come to test them. We are almost always only provided with one construct, and undoubtedly, careful construct design and testing would improve success rates; however, this would be a very resource-intensive exercise for target owners and is currently out of scope for our own activities. Interestingly, TSA has a considerably higher success rate at 64%, which likely reflects the technical simplicity of this technology and that it does not require covalent modification or immobilization of the protein. When looking at MST assay development success rates broken down by target class, we found the greatest success with cAMP binding proteins (100%) and protein–protein interaction targets (60%), but had less success with enzymes (38%) and protein–nucleic acid interaction targets (33%) (**Fig. 4B**). For this last class, we have been reliant on observing changes in MST upon binding of unlabeled oligonucleotides to labeled proteins to validate the assays, which has not been particularly successful. Interestingly, we often observe very good changes in MST by orientating the assay the other way around, that is, with unlabeled protein binding to labeled oligonucleotides. An assay set up in this format can be used to provide indirect evidence of target engagement by apparent competition of binding to the protein and is also very useful to identify RNA/DNA intercalators but does not provide direct evidence of target engagement, which is far more preferable. Usefully for us, targets that fail when using one technique typically work with one of the others, and overall we manage to enable 72% of the projects with one or more biophysical assays. The following are a couple of case studies detailing how MST has been applied to ELF projects.

## Case Study: Using MST and TSA to Triage Bacterial Kinase Screening Hits

The molecular target, a bacterial kinase that is essential for gram-negative bacteria survival with currently no known small-molecule inhibitors, was screened against the JECL comprising 445,917 compounds in a biochemical ADP Hunter Plus (DiscoverX, Fremont, CA) assay format. A total of 955 compounds, consisting of statistically significant hits from the HTS and potential false-negative compounds rescued using Bayesian modeling, were tested for confirmation of activity as dose–response curves. Of these, an IC_50_ of 20 µM or below was calculated for 73 compounds. To identify the most promising of the 73, they were tested for correct molecular identity and acceptable purity with liquid chromatography–mass spectrometry (LC-MS), and were also tested in TSA and MST.

**Figure 5.**
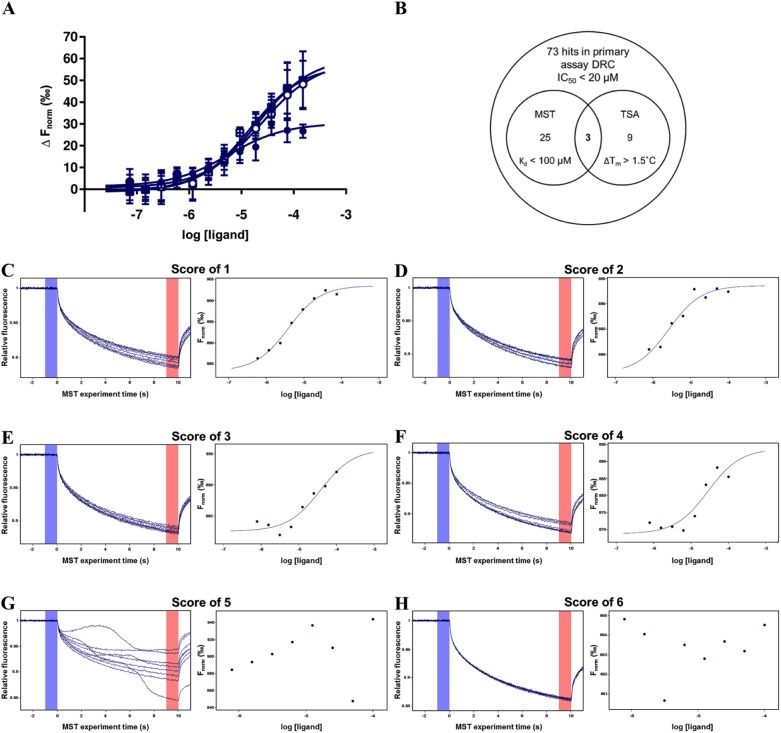
Case study 1: Hit selection for a bacterial kinase target using MST and TSA biophysical assays. (**A**) Normalized MST binding response (∆F_norm_) of phosphate acceptor substrate (150 µM to 73.2 nM) to 50 nM His tag–labeled protein kinase measured immediately (closed circles) and 30 min (open circles), 60 min (closed squares), and 120 min (open squares) after sample loading. (**B**) Venn diagram showing distribution and overlap of hits from the primary biochemical assay and MST and TSA biophysical assays. Representative MST traces (left panel) and normalized binding response (right panel) for compounds given scores of (**C**) 1, (**D**) 2, (**E**) 3, (**F**) 4, (**G**) 5, and (**H**) 6. DRC = dose–response curve.

For the MST assay development, the workflow described previously and shown in **Figure 2** was followed using the red dye/His tag labeling approach. A concentration of 50 nM His tag–labeled protein yielded a suitable fluorescent signal when applying 40% LED excitation power, 40% MST power, an MST on-time of 5 s, and an MST measurement time of 10 s. Slight protein adsorption was observed in standard capillaries, which was reduced with premium-coated capillaries. The buffer comprised 15 mM HEPES, 20 mM NaCl, 10 mM MgCl_2_, and 0.05% Tween 20 (pH 7.4), the same as that used in the primary assay minus bovine gamma globulin, to simplify the buffer, and EGTA, which cannot be used with His tag labeling due to the requirement for Ni^2+^ in the His–NTA interaction. The target protein was tolerant to ≤2.5% DMSO. No small-molecule reference was available to validate the assay, so binding of both the (nonpeptide) phosphate acceptor substrate and ATP was investigated. Binding of the phosphate acceptor substrate was measured at 0, 30, 60, and 120 min postloading into the capillaries to check that equilibrium was reached and ensure consistency and stability in data acquisition during a 45 min screening run. The data showed no sample adsorption onto capillaries over time, but the MST traces appeared less noisy after 30 min incubation than at time zero and the response amplitude was almost double (26.7 amplitude at time zero, 50.9 at 30 min, 54.8 at 60 min, and 53.6 at 120 min). The K_d_ of the substrate at time 0 min was 3.9 ± 1.9 µM (mean ± SD, *N* = 3), which is similar to the reported K_m_ of 6.8 µM; however, the consequence of the increased amplitude over time was that the K_d_ was three- to fivefold higher from 30 min onwards (18.2 ± 3.1 µM after 30 min, 17.1 ± 2 µM after 60 min, and 13.4 ± 1.9 µM after 120 min). This suggests that, at least for the phosphate acceptor substrate, it takes up to 30 min before the system reaches full equilibrium (**Fig. 5A**). Due to the difference in K_d_ values at 0 min and after 30 min, this ligand was used as a reference throughout screening at the start and at the end of each 45 min run. ATP binding was also investigated; however, no binding was apparent in a variety of buffers, in the presence of the phosphate acceptor substrate, when using ATP from at least three different suppliers or with Mg^2+^-ATP. This is in contrast to TSA, where shifts in the thermal melting temperature (T_m_) of +6 °C were observed for both substrates. This suggests that this MST assay may not be suitable for confirming the binding of orthosteric ATP-competitive inhibitors. In an ideal situation, this question about the sensitivity and specificity of the assay would be further addressed by using a range of reference compounds of various binding modes. This would provide the most comprehensive assessment of how the assay should be employed and how rigidly or not to interpret negative or positive results. Unfortunately, no reference compounds have been identified for this particular bacterial kinase to make such an assessment.

The screening hits were tested in MST at a single concentration of 40 µM in triplicate using the conditions established during assay development, with positive (substrate) and negative (DMSO) controls included at the start and at the end of each run. MST data were analyzed using the MO.Affinity Analysis software, and an unpaired *t* test confirmed that the normalized fluorescence signal (F_norm_) of the positive control (75 µM phosphate acceptor substrate) was statistically different from that of the negative control (1% DMSO), with a *p* value lower than 0.0001. A statistical cutoff of three standard deviations either side of the mean negative control was applied, with 36 out of 73 hits showing a positive MST response. The 36 compounds were subsequently tested as an 8-point twofold dilution series with a maximum concentration of 100 µM. The data for each compound were scored from 1 to 6 for likelihood of binding with 16 likely/possible binders scoring between 2 and 4, 5 aggregators scoring 5, and 15 nonbinders scoring 6 (**Fig. 5C–H**).

The strategy of testing all compounds with MST as a single concentration prior to determining the affinity of the actives was mentioned before and has been described in the literature.^32^ In that case, the compounds were tested in duplicate, as recommended by NanoTemper for single-point screening. Martin et al. screened 1700 fragments at a concentration of 500 µM and identified 124 hits with an MST signal out-with two standard deviations of the negative DMSO control;^32^ however, they did not confirm hit activity with full dose–responses in MST; rather, they validated 38 of the 124 hits in an orthogonal biophysical technique, heteronuclear single quantum coherence (HSQC) NMR. A second report of fragment screening using MST, from Linke et al., describes testing a library of 193 compounds as a 12-point twofold dilution series with a maximum concentration of 10 mM. They then retrospectively analyzed the ∆F_norm_ data for the 150 µM data point to estimate if single-concentration screening would have been sufficient to identify the hits. They concluded that this single-point screen would have been predictive in identifying the hits, which they later confirmed with x-ray crystallography.^31^ Our own experience has been slightly different in that only 21 of the 36 MST hits (58%) from the single-concentration screening strategy confirmed activity when tested as dilution series, despite having initially been tested in triplicate. This relatively low confirmation rate was a concern, so we tested the 37 “inactive” compounds as dilution series, 12 of which surprisingly appeared to be potential binders (32% scoring 1–4). It may have been that a cutoff of three standard deviations from the mean negative control was too stringent, so we reanalyzed the single-point data based on two standard deviations from the mean, which would have identified 6 of the 12. This still leaves six compounds, which we would class as being probable/potential binders, which were not identified using a single-concentration screening strategy and two standard deviation selection, providing a false-negative rate of 8%.

The 73 compounds were also tested in TSA at a single final concentration of 40 µM. Briefly, compounds were tested in the presence or absence of 1.36 µM target protein, with 1× Protein Thermal Shift dye (Applied Biosystems, Waltham, MA; excitation 570 nm, emission 591 nm) in assay buffer (15 mM HEPES, 20 mM NaCl, and 10 mM MgCl_2_ [pH 7.4]). Controls for “no protein” and 1% DMSO (corresponding to the final DMSO concentration found in the compound samples) were also included during screening. Protein unfolding was induced by increasing the sample temperature from 25 to 99 °C, with increments of 0.05 °C per second, a scan rate selected to maximize screening efficiency while not adversely affecting the quality of the data. Data were analyzed using the derivative method in the Protein Thermal Shift software from the QuantStudio 5 instrument (Applied Biosystems). A difference in target protein melting temperature in the presence of compound (ΔT_m_) of >1.5 °C was considered significant, as it was outside the 95% confidence intervals of the protein-/vehicle-only control, indicating that the compound had a stabilizing effect on the target protein and was likely to be a binder.

**Figure 5B** shows a Venn diagram representing the number of compounds reporting as active in the various assays. Three compounds were recognized as binders by both technologies. TSA identified nine compounds that significantly enhanced the stability of the target protein with ΔT_m_ values between 1.7 and 10.2 °C, but did not show a binding response in the MST assay. Conversely, 25 compounds that displayed a concentration-dependent MST binding response did not positively affect the target protein’s melting temperature in the TSA. Taken together, these results demonstrate that one biochemical and two biophysical techniques can identify distinct and overlapping populations of hits. Having this diversity of data allows for more informed decision making when selecting the final QHL, particularly when combined with the assessment of the compound structures, analytical results, and legacy screening data for the hits. The fact that hits from a biochemical screen produce variable responses in different biophysical assay formats will be familiar to most screening laboratories, and there are published examples of the phenomenon. Schiebel et al. describe the parallel screening of 361 fragments against endothiapepsin in four different biophysical techniques (MST, TSA, saturation transfer difference spectroscopy nuclear magnetic resonance [STD-NMR], and electrospray ionization mass spectroscopy [ESI-MS]), where they found a diversity of hit populations, with those identified by MST being more distinct than those identified in the other techniques.^23^ Martin et al. also found that their MST fragment screen on BRD9 identified a number of hits, later validated by NMR, which were not identified by DSF or SPR, indicating the importance of pragmatism and employing a diversity of techniques to inform hit selection.^32^ The process of validation of the hits from the bacterial kinase screen is currently ongoing, with resynthesis and testing with lower-throughout techniques such as NMR, ITC, and x-ray crystallography in an attempt to rationalize the different compound profiles across the different assay formats.

## Case Study: Using MST to Validate and Characterize Inhibitors of a Protein–Protein Interaction Target

As mentioned before, 15% of the ELF portfolio consists of protein-protein interaction targets that are typically difficult to drug due to the large, often quite shallow interaction surfaces between proteins, which can lead to a high proportion of false positives when screening.^54–56^ A protein-protein interaction target was screened against the JECL (318,132 compounds at that time) at 10 µM using an FP binding assay to measure the ability of compounds to compete with a fluorescently labeled peptide. From 1200 compounds initially identified as hits (hit rate = 0.38%) and 271 structurally similar compounds identified via Bayesian modeling, 377 compounds were confirmed as active. These were then tested for potency in the primary assay and for redox reactivity, a key criterion to avoid in the target product profile. Compounds showing redox activity or fluorescence interference in the FP assay were deselected, leaving 20 compounds that were submitted for analytical assessment, visual inspection, and legal clearance, resulting in a QHL of 8 compounds.

For this target, the rapid exclusion of interference or undesirable compounds during the screening cascade meant that biophysical assays were not required prior to nomination of the QHL; however, in a busy portfolio, with competing priorities and limited resources, committing to a program of chemistry requires convincing orthogonal evidence of target engagement. There were also questions raised around some of the compounds on the QHL, which contained a known fluorescent moiety, which it was felt may have an effect on the FP assay. We therefore established a label-free MST assay to characterize the QHL compounds. Pretests were performed using the unlabeled target protein and the UV detector to select a suitable concentration of labeled protein. Premium-coated and standard treated MST capillaries were compared to assess protein adsorption to the capillary walls. Unlabeled target protein (100, 200, and 500 nM) yielded signals of 2500 fluorescence units or more and did not show adsorption to either capillary type when using 50 mM HEPES buffer (pH 7.5) with 300 mM NaCl and 0.1% Pluronic F-127. Measurements were performed using 10% LED excitation power, 40% MST power, an MST on-time of 5 s, and an MST measurement time of 10 s. Those conditions were used in subsequent experiments with 150 nM protein. The label-free pretest also included assessing compound autofluorescence at the tryptophan wavelength, which identified UV autofluorescence for two of the QHL compounds.

**Figure 6.**
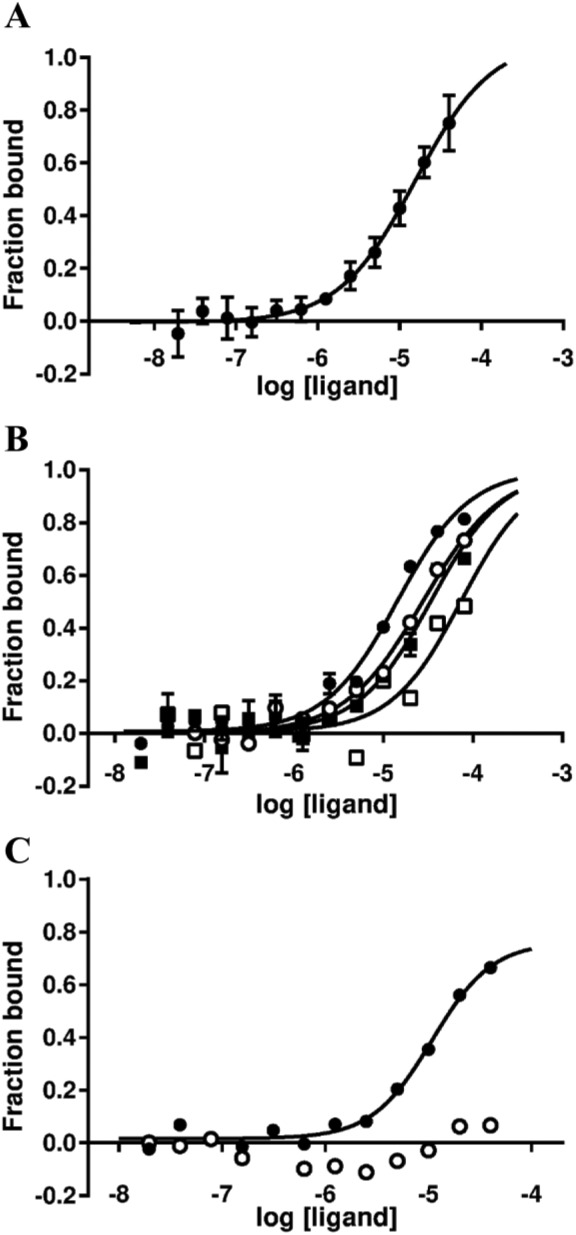
Case study 2: Hit characterization for a protein–protein interaction target. (**A**) MST binding response for one of the hit compounds from 40 µM to 19.5 nM compound vs. 150 nM unlabeled protein. Measurements were performed using the UV detector with 10% LED excitation power, 40% MST power, and 5 s MST on-time (K_d_ ± SD = 14.3 ± 3.0 µM). (**B**) Compound binding tested alone (closed circles) or with 3 µM (open circles), 10 µM (closed squares), or 30 µM (open squares) reference peptide. (**C**) The R-enantiomer binds (closed circles), while the S-enantiomer does not (open circles).

Of the eight QHL compounds, two structurally related hits showed a positive concentration-dependent binding response in the MST assay, five did not show any binding, and one displayed an aggregator profile. Both active molecules also showed a concentration-dependent decrease in initial fluorescence, meaning that the raw fluorescence data, and not the MST data, were analyzed to generate a binding curve (**Fig. 6A**). To ensure that this response was not due to a fluorescence artifact, particularly because these were the two compounds containing the known fluorescent moiety, it was essential to perform an SDS denaturation test. The MST signal of both compounds was abolished after denaturation of the target protein in 2% SDS/20 mM DTT and boiling for 5 min at 95 °C, indicating that the observed decrease in protein fluorescence was not simple fluorescence quenching, but rather a specific effect on the structurally intact protein.

Both compounds were resynthesized and their affinities in the FP and MST assays were in agreement with those obtained with the screening samples from the JECL. The addition of increasing concentrations of a peptide mimic of the target’s binding partner resulted in increasing K_d_ values for both hit compounds (**Fig. 6B**), confirming that they both bind at the interface of the protein-protein interaction. The two hit compounds were also clearly competitive with a literature reference small molecule. Both compounds contain a chiral center, and separation of the R- and S-stereoisomers showed that only the R-stereoisomer binds the target (**Fig. 6C**), providing chemical validation of the binding specificity and further evidence that the MST response was not due to fluorescence artifacts.

Taken together, these results provided enough confidence to initiate a chemistry program that resulted in the preparation of more than 110 new analogs and led to ligand-bound crystal structures, which helped rationalize the SAR. The success of this project can be significantly attributed to the positive MST data, as it is unlikely that chemistry resources would have been applied based solely on the screening data.

## Conclusions

In our experience, MST has proven to be a useful biophysical technique to apply to the triaging and characterizing of HTS hits. In the 2 years since implementing this technology, we have found that it has improved the chances of success for some programs by providing orthogonal evidence of target engagement that complements other biophysical techniques, such as SPR and TSA. On several programs, when SPR and TSA were unsuitable or the assay development was unsuccessful, MST provided the only corroborative orthogonal biophysical data. Conversely, MST assay development sometimes failed when SPR and/or TSA worked well. The reasons for the inconsistency in developing assays with different techniques are likely many and varied. In one example from the ELF, a target construct exhibited a relatively low melting temperature of 32 °C, indicating that it was quite an unstable protein. It is likely that from heating the sample within the capillaries, or potentially even at room temperature, a substantial amount of the protein would have been in an un- or misfolded state, which prevented us from observing ligand binding. Indeed, the results from SPR showed that at ambient temperature, it was difficult to resolve ligand binding, yet conducting it at 10 °C provided very good data. The temperature of the Monolith NT.Automated instrument can only be set to 25 °C, while the other NanoTemper Monolith instruments can be set to 25 °C or above, so the possibility of being able to lower the sample temperature may be useful in improving the success rate for developing assays when working with less stable proteins.

In our experience, a significant amount of the failure for MST (and SPR) assay development has been due to poorly validated reference molecules, particularly for new/difficult targets where high-quality, highly specific small molecules are not typically available. Ironically, this is one of the reasons that such targets are being screened within the ELF, to find new targeted chemical probes, but this can present a chicken-and-egg problem, where the validation of new assays requires highly validated reference molecules, yet the validation of new molecules requires highly validated assays. Therefore, the availability of multiple reference molecules, ideally small molecules, rather than endogenous protein or peptide ligands or substrates, is likely to improve the chance of successfully developing an MST assay and using it productively in confirming the target engagement of small-molecule hits from an HTS campaign. The other key components to successfully employing MST are protein quality and the ability to access multiple protein constructs, allowing for the evaluation of multiple labeling techniques. This is a significant limitation to the ELF workflow, as we principally rely on collaborators to provide the proteins. Another area for improvement of our workflow would be the integration of our MST instrument with an automated liquid handling system, such as that described by Sanofi and NanoTemper,^31^ thereby increasing the throughput, which might be able to more efficiently deal with the large numbers of hits that are identified when screening some targets.

As described here, we have observed some issues regarding the reliability of MST single-point screening. It appears that generating full dose–response curves is the most comprehensive way of identifying genuine binders, but this has a detrimental effect on throughput and screen cost. More study will be required to optimize a screening workflow, and we intend to investigate approaches such as varying the number of points within a dilution series and whether any other variables affect the likelihood of false negatives in single-point screening, such as incubation times, buffer composition, or target class.

Like other researchers, we find that MST is best used in combination with other biophysical techniques, as they all tend to identify different populations of hits with sometimes little overlap. For this reason, we would recommend using multiple assays and technologies during a screening campaign and assess hits within the context of the totality of the data, including biochemical and biophysical assay data, sample QC data, and structural assessment, when selecting the most promising hits.
